# Artificial Intelligence in Intracerebral Hemorrhage: Current Applications and Future Perspectives

**DOI:** 10.3390/jcm15145403

**Published:** 2026-07-10

**Authors:** Xinghua Xu, Jiashu Zhang, Zhichao Gan, Shiyu Zhang, Haoyang Zheng, Xiaolei Chen, Qun Wang

**Affiliations:** 1 Department of Neurosurgery, The First Medical Center of Chinese PLA General Hospital, Beijing 100853, Chinazsyflow@163.com (S.Z.); zhyer66@163.com (H.Z.);; 2 Medical School of Chinese PLA, Beijing 100853, China

**Keywords:** intracerebral hemorrhage, artificial intelligence, machine learning, deep learning, minimally invasive surgery

## Abstract

Intracerebral hemorrhage (ICH) remains one of the most severe forms of stroke and is associated with high mortality, poor functional outcomes, and substantial healthcare burden worldwide. Despite advances in neurocritical care and minimally invasive surgical techniques, the management of ICH remains challenging because of disease heterogeneity, rapid neurological deterioration, and the lack of effective individualized treatment strategies. In recent years, artificial intelligence (AI) has emerged as a promising tool for improving the diagnosis, prognostic evaluation, and precision management of ICH. A systematic literature search was performed in PubMed, Web of Science, and Embase to identify studies on AI applications in ICH, with predefined inclusion criteria focusing on imaging analysis, prognostic prediction, clinical decision support, and minimally invasive surgery. This review summarizes the major clinical applications, limitations, and future directions of AI in ICH, including multimodal foundation models, intelligent surgical assistance, and personalized precision care. Recent studies have demonstrated that AI-based models can significantly improve the accuracy of hematoma segmentation, hematoma expansion prediction, and functional outcome prognostication compared with conventional approaches. Despite encouraging progress, several important barriers continue to limit clinical translation, including data heterogeneity, limited external validation, insufficient interpretability, ethical and regulatory concerns, and challenges in workflow integration. Overall, AI has the potential to transform ICH management from conventional experience-based practice to data-driven, personalized, and precision neurosurgical care.

## 1. Introduction

Intracerebral hemorrhage (ICH) is among the most devastating forms of stroke and remains associated with substantial mortality and long-term disability worldwide [1,2]. Certain subtypes, such as lacunar syndromes (“hemorrhagic pure motor stroke”), are associated with a relatively favorable prognosis [3]; however, ICH as a whole remains a devastating condition. Despite advances in neurocritical care and minimally invasive surgical techniques, outcomes remain poor, largely due to hematoma heterogeneity, secondary brain injury, and the lack of individualized treatment strategies [4,5]. Traditional clinical assessment of ICH primarily relies on radiological interpretation, hematoma volume estimation, neurological examination, and physician experience [6,7,8]. However, these approaches are often limited by interobserver variability, delayed decision-making, and insufficient precision in predicting hematoma expansion, neurological deterioration, and functional outcomes. Acute lobar ICHs exhibit a distinct clinical profile and are associated with a more severe early prognosis compared with deep subcortical ICHs [9].

Recent advances in artificial intelligence (AI), particularly machine learning and deep learning technologies, have created new opportunities for improving multiple aspects of ICH management [10,11,12,13]. Increasing evidence suggests that AI may improve automated hematoma detection and segmentation, prognostic prediction, imaging biomarker analysis, and surgical decision support [10,12,13,14]. This review summarizes recent advances in AI-assisted imaging analysis, prognostic prediction, and neurosurgical applications in ICH and discusses current limitations and future directions for clinical translation.

To ensure comprehensive coverage of the literature, a systematic search was performed in PubMed, Web of Science, and Embase from inception to March 2026 to identify studies on AI applications in ICH. The search combined MeSH terms and free-text keywords related to ICH and AI. Additional studies were identified through reference screening.

## 2. Current Challenges in ICH Management

### 2.1. High Mortality and Disability

Despite advances in neurocritical care, imaging, and surgical techniques, the prognosis of ICH remains poor, with high mortality and substantial long-term disability [15,16]. Early neurological deterioration is often driven by rapid hematoma expansion, elevated intracranial pressure, and secondary brain injury, including perihematomal edema, neuroinflammation, oxidative stress, and blood–brain barrier disruption [17,18]. Survivors frequently experience persistent motor deficits, cognitive impairment, speech dysfunction, and reduced quality of life, resulting in substantial long-term functional dependence and caregiver burden. Clinical heterogeneity—including differences in hematoma location, volume, intraventricular extension, etiology, and patient comorbidities—further complicates prognosis and individualized treatment planning. While strategies such as blood pressure control, hemostatic therapy, minimally invasive surgery, and optimized neurocritical care have been explored [19,20,21], no universally effective management paradigm exists, underscoring the need for earlier diagnosis, refined risk stratification, and precision-based interventions.

### 2.2. Limitations of Traditional Assessment Methods

Current clinical assessment of ICH largely relies on neuroimaging, neurological examination, and physician experience. Non-contrast computed tomography (CT) remains the standard imaging modality for initial diagnosis, where key radiological features such as hematoma volume, location, intraventricular extension, and mass effect are assessed visually. Traditional volume estimation methods, such as the ABC/2 or Tada formula, are simple and widely used but may be inaccurate in irregularly shaped or multilobulated hematomas [22]. Manual segmentation is labor-intensive, operator-dependent, and often impractical in emergency settings. These limitations contribute to interobserver variability, delayed decision-making, and imprecise prediction of hematoma expansion and clinical outcomes, highlighting the need for automated and AI-assisted quantitative imaging analysis.

### 2.3. Challenges in Surgical Decision-Making

Surgical management of ICH remains controversial despite decades of clinical trials and observational studies [15,23]. Minimally invasive hematoma evacuation may benefit selected patients, but optimal strategies depend on multiple factors, such as hematoma location and volume, timing of intervention, patient age, baseline neurological status, intraventricular involvement, and comorbidities. The ideal timing of surgery is uncertain: ultra-early evacuation may increase the risk of rebleeding, whereas delayed intervention may result in missed opportunities to prevent secondary injury [24]. Current guidelines provide limited recommendations for individualized surgical planning [15], and treatment decisions still largely rely on clinician experience and empirical judgment rather than data-driven approaches.

### 2.4. Lack of Precision and Individualized Treatment

ICH is highly heterogeneous, yet current management remains largely non-individualized and protocol-driven. Patients with similar hematoma volumes may follow markedly different clinical courses due to variations in hematoma location, vascular pathology, genetic background, inflammatory response, and systemic physiology [25]. Existing guidelines, derived from population-level studies, often fail to account for patient-specific characteristics and the dynamic evolution of hematoma-related secondary injury over time [26]. Consequently, accurate prediction of hematoma expansion, neurological deterioration, and functional outcomes remains challenging, and therapeutic strategies are seldom truly personalized in routine clinical practice. Integration of multimodal clinical data is still fragmented and underutilized, leaving a gap for precision medicine approaches.

## 3. Fundamentals of AI in Medicine

AI encompasses computational approaches capable of learning from data, recognizing patterns, and supporting prediction or decision-making in clinical medicine. Advances in computational power, medical imaging, and big data analytics have accelerated the integration of AI into healthcare [27,28,29]. In ICH, AI is increasingly applied to support imaging interpretation, risk prediction, and precision management. Key AI methodologies include machine learning, deep learning, and radiomics, which form the technical foundation for most current clinical applications. In general, AI systems in medicine follow a structured workflow consisting of data acquisition, preprocessing, feature extraction or representation learning, model training, validation, and clinical inference, ultimately enabling decision support or automated analysis in clinical practice. Understanding these key AI methodologies is essential for appreciating their current and potential clinical applications.

### 3.1. Machine Learning

Machine learning (ML) is a core branch of AI in which algorithms learn patterns from data to make predictions or decisions without explicit programming. In clinical settings, ML models are trained on structured datasets—including demographic, laboratory, imaging, and physiological variables—to predict outcomes such as hematoma expansion, neurological deterioration, or functional recovery [13,30]. Compared with traditional statistical scoring systems, ML can model complex nonlinear relationships and identify high-risk patient subgroups [31,32]. Typical ML pipelines involve manual or semi-automated feature engineering followed by model training using classifiers such as logistic regression, random forest, or support vector machines. Limitations include reliance on manually engineered features and sensitivity to dataset quality.

### 3.2. Deep Learning

Deep learning (DL) is a specialized subset of ML that employs artificial neural networks with multiple layers to automatically learn hierarchical feature representations from raw data [33]. Convolutional neural networks (CNNs) are widely used for image classification, segmentation, and pattern recognition in neuroimaging. Transformer-based architectures, capable of modeling long-range dependencies, have recently enabled the integration of multimodal information from imaging, clinical records, and laboratory data. DL models demonstrated excellent performance in automated hematoma segmentation, perihematomal edema quantification, and hematoma volume estimation [34]. By analyzing large-scale imaging and clinical datasets, DL systems can identify subtle imaging patterns associated with hematoma expansion, neurological deterioration, or long-term functional outcomes [35,36]. Unlike traditional ML, DL eliminates the need for manual feature engineering by directly learning hierarchical representations from raw imaging or clinical data through backpropagation-based optimization. However, DL models typically require large, annotated datasets and suffer from limited interpretability, commonly referred to as the “black-box” problem.

### 3.3. Computer Vision and Large Language Models

Computer vision (CV) applies AI techniques to analyze visual data. In medicine, CV is used for automated detection, segmentation, and quantification of lesions on imaging studies. For ICH, CV enables rapid identification of intracranial hemorrhage, hematoma volume estimation, perihematomal edema quantification, and recognition of imaging biomarkers. CV also supports intraoperative navigation, trajectory planning, instrument tracking, and 3D reconstruction, enhancing precision in minimally invasive procedures. In clinical imaging workflows, computer vision systems typically operate through image acquisition, preprocessing, object detection or segmentation, and quantitative output generation for clinical interpretation.

### 3.4. Large Language Models

Large language models (LLMs) process and interpret natural language data at scale. In clinical contexts, LLMs can summarize radiology reports, extract key information from electronic health records, assist decision-making, and facilitate guideline retrieval. Multimodal LLMs that combine text, imaging, and physiological data may further support integrated clinical reasoning and risk assessment. Rigorous validation and clinician oversight are essential to ensure safety, accuracy, and compliance with medico-legal and privacy standards. From a technical perspective, LLMs are trained on large-scale text corpora using self-supervised learning to predict token sequences, enabling them to capture semantic and contextual relationships in medical language.

### 3.5. Multimodal AI

Multimodal AI refers to systems that integrate diverse data sources, such as imaging, clinical records, laboratory results, genomics, physiological monitoring, operative videos, and natural language text. By capturing complex interactions between heterogeneous data, multimodal AI can provide a more comprehensive understanding of disease processes and improve individualized prediction and treatment planning [37]. In ICH, this approach has the potential to support intelligent stroke platforms capable of real-time monitoring, dynamic risk assessment, and clinician-supervised decision support, facilitating a transition toward more personalized and data-driven care. Multimodal learning typically involves feature alignment and fusion strategies, such as early fusion, late fusion, or attention-based fusion, to integrate heterogeneous data representations into a unified predictive model.

## 4. AI in ICH: From Imaging to Precision Surgery

AI is transforming ICH management across diagnosis, prognostic assessment, and precision-guided surgery. Unlike traditional approaches that rely heavily on physician experience, AI can process large-scale heterogeneous datasets, identify complex nonlinear relationships, and provide rapid quantitative analysis. Recent advances in machine learning, deep learning, computer vision, and multimodal AI enable increasingly sophisticated applications in imaging interpretation, outcome prediction, surgical planning, and minimally invasive neurosurgery. The representative AI applications in ICH management were summarized in Table 1. Overall, these applications collectively support a continuous clinical workflow spanning early diagnosis, risk stratification, perioperative decision-making, and postoperative outcome monitoring. Notably, although most existing studies report consistently high performance metrics, direct comparison across studies remains challenging due to differences in dataset size, imaging protocols, outcome definitions, and validation strategies.

### 4.1. AI in Neuroimaging Analysis

Neuroimaging is central to ICH diagnosis, risk stratification, treatment planning, and follow-up. Rapid and accurate hemorrhage detection is critical in hyperacute settings. AI-based imaging analysis, particularly CNNs, has demonstrated high sensitivity and specificity for detecting ICH on non-contrast CT and differentiating it from ischemic stroke, calcifications, or artifacts [10,39]. AI-assisted triage can accelerate workflow, reduce dependence on immediate radiologist availability, and facilitate early detection of subtle or small-volume hemorrhages. Compared with labor-intensive and observer-dependent manual delineation, these models provide more efficient and reproducible boundary segmentation. Emerging approaches further extend to quantifying perihematomal edema, extracting radiomics features, and characterizing hematoma shape and texture, which are associated with expansion risk, neurological deterioration, and functional outcomes. Yu et al. demonstrated that the UNet achieved expert-level performance in hematoma segmentation on CT scans (Dice score: 0.869) and showed excellent agreement with manual hematoma volume measurements (R^2^ = 0.9979) [11]. The integration of multimodal imaging data—including CT, MRI, perfusion imaging, and clinical data—further improves individualized risk stratification and precision treatment planning.

### 4.2. AI for Prognosis and Outcome Prediction

Accurate prediction of hematoma expansion, mortality, and functional outcomes is essential for individualized ICH management. Although conventional scoring systems are widely used, they often fail to capture complex interactions among imaging, clinical, and laboratory data. AI models can integrate multidimensional data to predict individual risk more accurately [13,31,36,38]. Hematoma expansion occurs in 20–40% of patients during the hyperacute phase and is strongly associated with poor outcomes. AI models incorporating imaging data and clinical features can stratify patients for intensive monitoring, hemostatic therapy, or early surgical intervention [32,35]. Mortality prediction assists triage and resource allocation, while functional outcome prediction, commonly assessed via the modified Rankin Scale (mRS), guides rehabilitation and long-term care planning. Xu et al. found that the random forest and XGBoost models achieved the best performance in predicting 6-month functional outcomes, with accuracies exceeding 90% [13]. Dynamic AI models can update risk predictions using longitudinal data, enabling adaptive treatment strategies.

### 4.3. AI in Surgical Decision-Making and Precision Treatment

ICH surgical management requires careful patient selection, timing, and individualized strategy. AI-based decision-support systems can analyze multimodal clinical data to guide objective and evidence-based decisions. Predictive models assist in identifying patients most likely to benefit from surgery, optimizing timing, and evaluating procedural risk. Preoperative planning may incorporate imaging-based trajectory optimization to minimize injury to eloquent cortex and deep perforating vessels. Although still investigational, real-time intraoperative AI guidance holds potential for detecting residual hematoma, monitoring catheter position, and assisting dynamic decision-making.

### 4.4. AI-Assisted Minimally Invasive Neurosurgery

Minimally invasive hematoma evacuation is increasingly preferred for its lower morbidity and faster postoperative recovery [21,40]. The integration of AI further enhances procedural precision and standardization. Stereotactic planning systems optimize trajectory design and catheter placement while minimizing injury to eloquent brain regions and critical vasculature. Robotic-assisted platforms provide stable and reproducible instrument control, reducing tremor and improving targeting accuracy. Computer vision enables real-time anatomical recognition, instrument tracking, and augmented reality–based navigation, supporting more precise operative guidance. Gan et al. developed an AI-driven ICH surgical planning system with clinical-grade segmentation (Dice score: 0.90) and demonstrated safe trajectory planning in >95% of supratentorial cases, supporting more standardized and accessible neurosurgical decision-making [14]. In addition, AI-assisted technologies facilitate surgical training through virtual simulation, performance assessment, and intelligent navigation, improving technical proficiency. Despite these advantages, clinical translation remains limited by high development costs, technical complexity, regulatory constraints, and medico-legal considerations. Overall, the convergence of AI, robotics, computer vision, and advanced visualization technologies is progressively reshaping minimally invasive ICH surgery and supporting more standardized and precise operative workflows.

## 5. Current Limitations and Challenges

Despite rapid advances in AI for ICH, significant challenges remain before these systems can be reliably translated into routine clinical practice. Current limitations span data quality and generalizability, model interpretability, clinical integration, and ethical governance. A major barrier is the substantial heterogeneity of ICH, which encompasses variability in hematoma location, volume, etiology, imaging appearance, and treatment strategies. This intrinsic biological variability is further amplified by differences in imaging protocols, scanner types, acquisition parameters, and institutional workflows, resulting in significant domain shifts across datasets. In addition, hematoma location may influence imaging features and radiological heterogeneity in ICH. It may also affect the performance and generalizability of prognostic prediction models. Clinical variability—including differences in comorbidities, blood pressure management, surgical approaches, and perioperative care—further complicates data standardization and limits reproducibility across institutions. Collectively, these factors substantially impair model generalizability in real-world settings.

Compounding this issue, many existing AI models are developed on relatively small, single-center retrospective datasets, with limited external validation. Such datasets are often insufficient to capture the full spectrum of disease heterogeneity, leading to model instability and potential overfitting. As a result, performance observed in internal validation frequently fails to translate consistently to independent or multi-center cohorts, particularly in underrepresented scenarios such as infratentorial hemorrhage, pediatric cases, or complex postoperative conditions. Another critical limitation is the lack of interpretability in many deep learning models, commonly referred to as the “black-box” problem. The absence of transparent decision pathways reduces clinician trust and constrains adoption in high-stakes neurosurgical decision-making. Moreover, models may inadvertently learn spurious correlations or institution-specific patterns, reflecting hidden biases within training datasets rather than true pathophysiological relationships.

In parallel, ethical, legal, and governance issues remain unresolved. The absence of clear medico-legal frameworks for shared human–AI responsibility may limit adoption in high-risk neurosurgical settings. Key concerns include patient privacy and data security, particularly given the reliance on large-scale multimodal datasets. Algorithmic bias may also result in performance disparities across populations, raising concerns regarding fairness and equity. Furthermore, accountability in AI-assisted decision-making remains unclear in cases of diagnostic error or adverse outcomes. These systems should therefore be regarded strictly as clinical decision-support tools, with final responsibility remaining with the treating physician, supported by adequate transparency and oversight frameworks.

Finally, automation bias represents an important human-factor limitation. Clinicians may over-rely on AI-generated outputs, potentially weakening independent judgment and error detection. This risk is particularly relevant in neurocritical care, where rapid decision-making is required. Accordingly, AI should be used to augment rather than replace clinical reasoning, and all outputs must be interpreted within the context of comprehensive clinical evaluation.

## 6. Future Perspectives

Future advances in ICH management are likely to be driven by the convergence of AI, multimodal data integration, and intelligent robotic technologies. Future AI development in ICH is expected to increasingly focus on explainable, clinically interpretable, and workflow-integrated decision support across diagnosis, prognostic assessment, surgical planning, and treatment guidance. While current applications focus on isolated tasks such as imaging analysis or outcome prediction, future systems are likely to evolve into integrated platforms supporting the entire clinical workflow.

Multimodal foundation models can process diverse data—including imaging, clinical records, laboratory tests, operative videos, and text—enabling comprehensive risk assessment and personalized treatment planning. Beyond static assessment, future models may incorporate longitudinal temporal data, allowing dynamic prediction of hematoma expansion, perihematomal edema progression, and treatment response. Large language and multimodal AI may further assist documentation, guideline retrieval, and decision support with proper validation and clinician oversight. In addition, privacy-preserving learning frameworks may facilitate large-scale multicenter collaboration while reducing the need for centralized data sharing, thereby improving model generalizability and robustness.

AI-enabled intelligent surgical navigation and robotic systems may further enhance minimally invasive hematoma evacuation by improving precision, reproducibility, and procedural safety. Real-time intraoperative AI can support anatomical landmark recognition, trajectory optimization, catheter placement, intraoperative adaptation, and residual hematoma detection. Integration with computer vision, augmented reality, and robotic assistance may enable more adaptive and personalized surgical interventions. Current applications remain largely assistive, requiring further technical, ethical, and regulatory development. Digital twin simulations offer the potential to model hematoma evolution, edema progression, and tissue deformation, supporting individualized preoperative planning, though this approach remains exploratory. Intelligent stroke systems may integrate AI, multimodal monitoring, and robotics to support triage, imaging interpretation, risk prediction, surgical planning, and rehabilitation.

**Figure 1:**
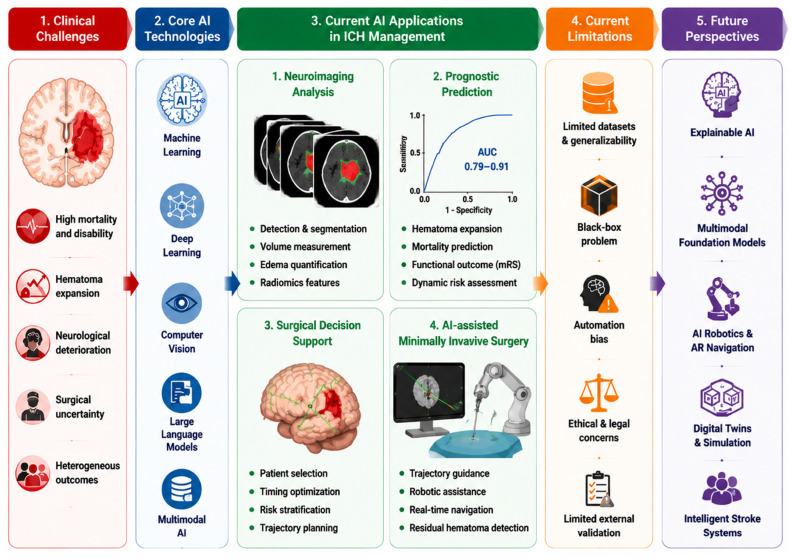
Overview of current applications and future perspectives of AI in ICH. The figure summarizes major clinical challenges of ICH, core AI technologies, current clinical applications, existing limitations, and future directions toward human–AI collaborative precision care.

Successful clinical translation will depend on prospective validation, standardized datasets, workflow integration, regulatory approval, and clinician trust, as well as real-world implementation studies. Attention to ethics, privacy, fairness, and accountability will remain essential. From a medico-legal perspective, while regulatory frameworks for AI-assisted decision-making continue to evolve, the final clinical decision-making responsibility must remain with the treating physician. Ultimately, the greatest value of AI in ICH will lie not in autonomous decision-making, but in augmenting clinician judgment to enable more precise, timely, and individualized care. The major clinical challenges, core AI technologies, current applications, and future perspectives of AI in ICH are summarized in Figure 1.

## 7. Conclusions

ICH remains one of the most severe forms of stroke, with persistent challenges in diagnosis, prognostication, and treatment decision-making. AI offers new opportunities to enhance ICH care through imaging analysis, outcome prediction, and AI-assisted minimally invasive surgery. However, translation into routine clinical practice remains limited by data heterogeneity, weak external validation, limited interpretability, and incomplete integration into clinical workflows. Progress will depend on large-scale multicenter collaboration and prospective, clinically grounded validation frameworks. More fundamentally, AI is likely to transform ICH management from isolated predictive modeling toward integrated, continuously adaptive decision-support systems embedded within real clinical workflows. With responsible development and validation, AI may enable a shift toward more precise, personalized, and data-driven neurosurgical care for patients with ICH.

## Figures and Tables

**Table 1:** Representative AI applications in ICH management.

Author (Year)	Study Design	Sample Size	AI Technique	Clinical Task	Performance Metrics
Dhar R (2020) [10]	Prospective	124	Convolutional neural network (CNN)	Volumes of hemorrhage and PHE	Dice score: 0.90 for hemorrhage and 0.54 for perihematomal edema
Xu X (2021) [13]	Retrospective	270	Machine learning	Prognostic prediction	Sensitivity 93.3%, specificity 92.5%
Yu N (2022) [11]	Retrospective	512	Dimension reduction UNet deep learning	Hematoma segmentation and volume measurement	Dice score: 0.861, R^2^ = 0.9979
Tanioka S (2022) [32]	Retrospective	422	Machine learning	Hematoma expansion prediction	AUC: 0.790
Tong G (2023) [35]	Retrospective	351	3D U-Net deep learning	Hematoma segmentation	Accuracy 96%
Kok YE (2022) [34]	Retrospective	1732	3D no-new-U-Net deep learning	Hematoma and PHE segmentation	Dice score: 0.92, 0.66, and 1.00 for ICH, PHE, and IVH
Chen Y (2023) [30]	Retrospective	550	Machine learning	Predict PHE expansion	AUC: 0.840 and 0.705 for early and delayed PHE expansion
Zhao X (2024) [12]	Retrospective	604	Convolution model and XGBoost	Functional outcome prediction	AUC: 0.829, 0.871, and 0.905 for convolution, XGBoost, and fusion models
Geng Z (2024) [38]	Retrospective	413	Machine learning	90-day prognostic outcome	AUC: 0.817
Gan Z (2024) [14]	Retrospective	347	nnU-Net deep learning	Hematoma segmentation and trajectory planning	Dice score: 0.90 for hematoma segmentation, low-risk trajectory in 80.8%
Wei L (2025) [31]	Retrospective	1091	Machine learning	90-day prognosis outcome	Fusion feature model AUC: 0.796
Chen Y (2025) [36]	Retrospective	882	Deep learning	Outcome prediction	C-index: 0.742, 0.712, and 0.779 for dependent living, disability, and severe disability

AUC: area under the curve; ICH: intracerebral hemorrhage; IVH: intraventricular hemorrhage; PHE: perihematomal edema.

## Data Availability

The data that support the findings of this study are available from the corresponding author upon reasonable request.
